# Secured remote health monitoring system

**DOI:** 10.1049/htl.2017.0033

**Published:** 2017-09-14

**Authors:** Duraisamy Sathya, Pugalendhi Ganesh Kumar

**Affiliations:** 1 Department of Computer Science and Engineering, Kumaraguru College of Technology, Coimbatore, Tamilnadu, India; 2 Department of Information Technology, Anna University Regional Campus, Coimbatore, Tamilnadu, India

**Keywords:** patient monitoring, wireless sensor networks, biomedical measurement, secured remote health monitoring system, wireless medical sensor network, symmetric algorithm, attribute-based encryption, data transmission

## Abstract

Wireless medical sensor network is used in healthcare applications that have the collections of biosensors connected to a human body or emergency care unit to monitor the patient's physiological vital status. The real-time medical data collected using wearable medical sensors are transmitted to a diagnostic centre. The data generated from the sensors are aggregated at this centre and transmitted further to the doctor's personal digital assistant for diagnosis. The unauthorised access of one's health data may lead to misuse and legal complications while unreliable data transmission or storage may lead to life threatening risk to patients. So, this Letter combines the symmetric algorithm and attribute-based encryption to secure the data transmission and access control system for medical sensor network. In this work, existing systems and their algorithm are compared for identifying the best performance. The work also shows the graphical comparison of encryption time, decryption time and total computation time of the existing and the proposed systems.

## 1 Introduction

Wireless sensor network (WSN) is an upcoming technology in existing researches, which makes the human life more comfortable. A wireless sensor is the smallest entity of a network and it is used in many applications such as the armed forces, water irrigation, testing soil moisture, structural health monitoring, field monitoring, volcanic activity monitoring, human health care monitoring and so on.

The recent progress in WSNs has given rise to its numerous application areas in healthcare. It has created a new field of wireless medical sensor networks (WMSNs). Using any of the wearable and non-wearable biosensor devices, human health can be tracked and monitored. WMSN is used to monitor the sports person's health activities and to record the patient's health status, who needs continuous health monitoring either in hospital or at home [1]. The data collected through biosensors are transmitted over wireless network to the diagnostic centre. The transmission of health data through wireless networks is susceptible to attacks. During transmission, the person's data may be misused by others and it may create a danger to the person's life [2]. Therefore, security is a principal requirement of healthcare applications.

The security mechanisms applied in traditional network cannot be applied to WMSN because of the resource constraints of sensor network [3] such as low computational capacity, battery powered device, and broadcast communication in nature, but WMSN's security needs are the same as that of conventional networks in terms of network availability, authenticity, confidentiality, data freshness and integrity. Many researchers have explored the possibility of providing the security to medical data. Some of the works have concentrated on providing the integrity and confidentiality of data alone and others have contributed only to providing authentication of data [4]. In the proposed secured remote health monitoring system (SHS), both the encryption algorithm and access control mechanisms are combined together to provide integrity, authenticity and confidentiality of data.

In the present work, blowfish encryption is used to transmit the examined medical data securely to the server of diagnostic centre. The data generated from the sensors are aggregated at this centre and transmitted further to the doctor's personal digital assistant (PDA). The data from the diagnostic centre must be accessed by the authorised doctors, nurses and technicians in hospitals. So, attribute-based encryption (ABE) algorithm is also implemented over the network. If an abnormality is detected in a patient's data, medical professionals at healthcare centre will quickly react to patients' emergency condition and save their lives by sending ambulance and medical people to the patient's location. The experimental results demonstrate the efficiency of the proposed system through its comparison with similar approaches.

The rest of this Letter is organised as follows. Section 2 describes concise overview of the related works, Section 3 presents the performance comparison of symmetric algorithms, Section 4 explains the proposed system, Section 5 presents experimental results and finally Section 6 gives the conclusion.

## 2 Information on related works

In [5], a novel and lightweight system to secure WMSNs has been proposed, which involves four segments: (i) the system initialisation segment where the network server builds up a medical sensor network (MSN), (ii) the user joining segment where the user can interact with the MSN via commands, (iii) in the regular user segment, the medical data from each biosensor node is transmitted to the network server via the controller (mobile phones), (iv) in the user command segment, the network user can construct the new command and it can be sent to the network server through the proxy signature key. The system achieves secure transmission and access control by using advanced encryption standard (AES) encryption algorithm and message digest 5 (MD5) algorithm, a one-way hash function. It does not use any verification table at the server which reduces storage space and computational power.

To provide data store and data access security, the data collected over the medical sensors are splitted into three components and stored in three distributed servers [6]. For example, the sensed medical data *ρ* is splitted into three integers *α*, *β*, *γ* such that *α* + *β* + *γ* = *ρ*. These *α*, *β*, *γ* values along with personal identification attributes are stored in three distributed servers S1, S2, S3 as {*A_i_*, *α_i_*}, {*A_i_*, *β_i_*} and {*A_i_*, *γ_i_*}, respectively. The Paillier cryptosystem and digital signature are used for providing access control and privacy to the medical data. The advantage of the method is that it is secure against outsider and insider attacks due to distributed servers and the value of *γ* can only be computed by knowing *α* and *β* values.

An alternative to the cryptographic techniques, the biological traits can be used to protect the personal health information. In the system [7], the biometric approach is used to secure the keys and to identify sensor motes in the network. The inter-pulse interval (IPI) of heart beat is calculated from electrocardiogram (ECG) and photoplethysmogram signals. From the IPI, the binary entity identifier is generated, which is used to identify the sensor motes in the body sensor network.

In [8], the body sensor nodes are used to monitor the patient's physiological signals like ECG signal, glucose level, blood pressure and body temperature. These readings are transmitted to the patient's PDA via Bluetooth. In the PDA, the steganography technique is used to hide the patient's information. As a result, a watermarked ECG signal is transmitted to the hospital via internet. The system provides data authentication, i.e. only the authorised doctors can access the patient's information and others can see only the watermarked signals.

In [9], network architecture consists of three tiers of network. (i) The sensor network tier consists of wearable sensor network to monitor the physical information. (ii) The mobile computing network tier consists of PDA and laptop to route the medical data to the remote base station or server. (iii) The back-end network tier consists of fixed stations and servers to process the sensed data received from mobile computing devices and it stores the data for future purposes. The Bluetooth secure protocol and public key infrastructure based cryptography are utilised to secure the transmission of data among the sensor nodes, mobile nodes and server.

In [10], the body area network collects the body parameters or body movements and transmits them to data sink (mobile device). The system follows four steps: step 1 presents the system initialisation which shares the public parameters and master key to all nodes in the network. Step 2 generates private keys based on the user attributes, which will be used to decrypt the ciphertext if the attributes satisfy the access tree. Step 3 encrypts the session key using ciphertext policy ABE (CP-ABE) and encrypts the message using AES. In step 4, the data consumers (doctors, nurses) decrypt the data to gain session key and by using the session key, they decrypt the ciphertext. The system provides security for data consumers, data sink and sensors, but the storage and computation cost of the method is higher.

## 3 Performance comparison of symmetric key algorithms

The symmetric key algorithms have very small key size, less memory usage and less computation time compared with asymmetric key algorithms. Further, the symmetric key algorithms are suitable for communication between small numbers of users [11] since the medical data would be communicated only to a few users like doctors, nurses, technicians and relatives of the patient. In SHS, the symmetric algorithms are chosen as the suitable algorithms for providing privacy to medical data.

The symmetric key encryption uses the same key for both encryption and decryption, whereas asymmetric key algorithms use different keys for encryption and decryption. The symmetric key algorithms are further divided into two types: stream and block cipher [12]. The stream ciphers usually encrypt bit by bit and for each bit encryption, they use different keys. The block ciphers are used to encrypt the block of data or files (64–128 bits in size) and the same key is used for encrypting each of the blocks [13]. The patient is monitored for a period of time and his or her health data are transmitted as a block or file to the hospital. In SHS, the medical data are encrypted using symmetric block cipher.

The most common symmetric algorithms, namely AES, data encryption standard (DES), Rivest's cipher 6 (RC6), blowfish and International data encryption algorithm (IDEA) are considered for performance comparison. The Java is used for implementing the algorithms over the dataset referred in [14]. The dataset have 130 observations and three variables which contain normal body temperature, gender and heart beat rate. The performance comparisons of symmetric algorithms are carried out on the basis of encryption cost, decryption cost and total computation time.

Table 1 shows the encryption time, Table 2 shows the decryption time while Table 3 shows the total computation time of the algorithms which include key generation, encryption and decryption time. From the performance analysis, the blowfish has been found to have very less encryption and decryption cost compared with the other algorithms. Since the medical data need to be transmitted within a short span of time, the blowfish algorithm is used for providing privacy in SHS.

**Table 1:** Encryption time of symmetric algorithms

	51 kB	204 kB	407 kB	1531 kB
AES, s	1	2	3	8
DES, s	0.5	0.98	2	7
blowfish, s	0.23	0.67	1.5	3
RC6, s	20	55	123	233
IDEA, s	0.2	0.56	2	3.5

**Table 2:** Decryption time of symmetric algorithms

	51 kB	204 kB	407 kB	1531 kB
AES, s	3	5	7.6	8.23
DES, s	2	4.5	6	7.6
blowfish, s	1	2.3	3.5	5.6
RC6, s	100	134	233	435
IDEA, s	1.2	1.5	2.3	4.5

**Table 3:** Total computation time of symmetric algorithms

	51 kB	204 kB	407 kB	1531 kB
AES, s	8.7	10.6	15.6	20
DES, s	7	9	14	19
blowfish, s	5	7	8	11
RC6, s	200	232	345	678
IDEA, s	2	5	6	10

## 4 Proposed system

**Fig. 1:**
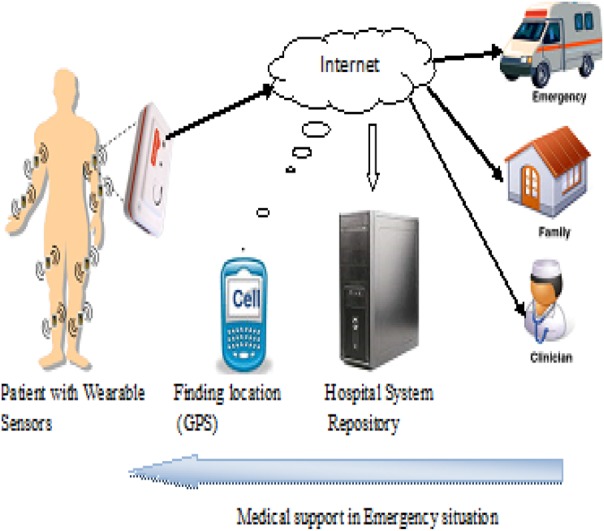
Model of the proposed system

One or more sensors like heart rate sensor, blood pressure sensor, ECG sensor and body temperature sensor can be connected to a patient's body based on the need. The blowfish algorithm is used to encrypt the sensor data on the common controller unit (mobile phone) before any transmission. Wi-Fi or 3G technology can be used to transmit the information, which is in encrypted form to the diagnostic centre. The data that come from the sensors are aggregated at this centre. The database is created at the diagnostic centre, and this can log the information received and help to monitor the patient's health status. ABE technique is used to access the medical data by authorised doctors, nurses and technicians. If doctors' diagnosed patient is in abnormal condition, then the ambulance will fly to the patient's location by the use of global positioning system. The proposed system is illustrated in Fig. 1.

### 4.1 Blowfish algorithm

In the current work, the blowfish algorithm is used to encrypt the medical data and these encrypted medical data are transmitted to the diagnostic centre. The blowfish algorithm uses variable key length from 32 to 448 bits and 64-bit block size. It is a 16-round Feistel cipher which uses large key dependent S-boxes [15].

**Fig. 2:**
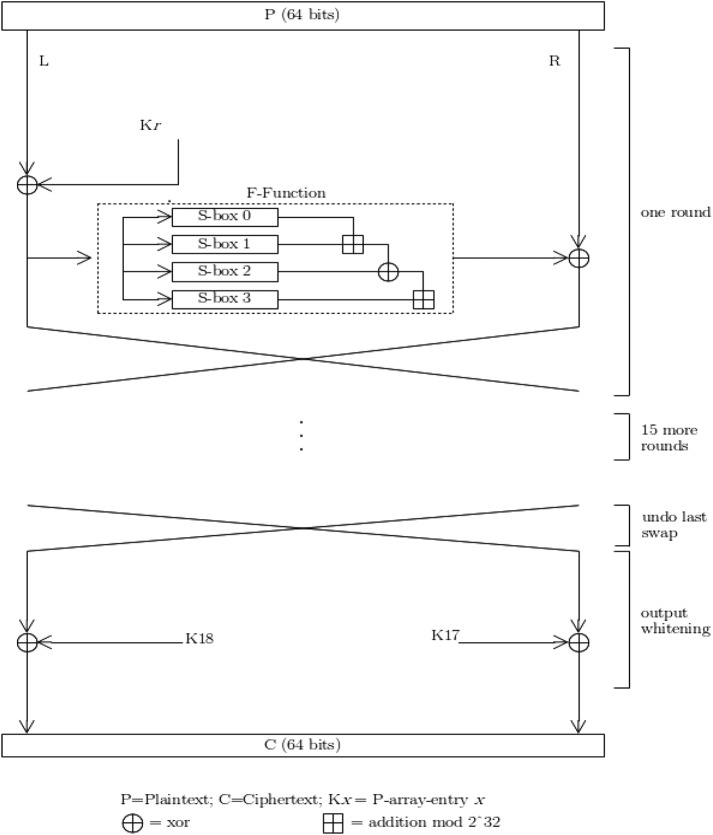
Blowfish encryption routine

Fig. 2 shows blowfish encryption algorithm routine. Each line corresponds to 32 bits. It has five sub-key arrays: four 256-entry S-boxes (S-box 0, S-box 1, S-box 2, S-box 3) and one 18-entry P-array (denoted as *K*).

Every round (*r*) consists of four steps.

- The left half (L) of the data are XORed with the *r*th P-array entry.
- Blowfish's *F*-function uses the XORed data as input.
- The *F*-function's output are XORed with the right half (R) of the data.
- Left half (L) and right half (R) are swapped.

The *F*-function divides the 32-bit input into four eight-bit quarters, and these quarters are used as input to the S-boxes. The S-boxes accept input of size 8-bit and produces output of size 32-bit. The outputs are added modulo 2^32^ and XORed to produce the final output of size 32-bit. After the 16th round, undo the last swap, and XOR R with K17 and L with K18. Decryption process is similar to the encryption process, except that P1, P2,...., P18 is used in the reverse order.

### 4.2 Ciphertext policy attribute based encryption

The medical data would be accessed by many users like relatives, doctors, nurses and technicians from the hospital. As the medical data need to be accessed only by the authorised users, the ciphertext policy access control is implemented at the centre [16]. In the ciphertext policy, encryption is done with the public key parameters, the set of attributes and a message. The private key of each user is associated with a set of attributes representing that user's permissions. Only the users tied to the relevant attributes can decrypt the message.

The CP-ABE algorithm consists of four steps [17]:

- Setup (*λ*, *U*) → (PK, MK): In this, *U* is the universe description that defines the set of allowed attributes in the system. The setup algorithm combines a security parameter *λ* and a universe description *U* to generate the output, the master secret key MK and the public parameters PK.
- Encrypt (PK, *S*, *M*) → CT: The encryption algorithm takes the combination of the public parameters PK, a set of attributes *S*, and a message *M* as input and outputs a ciphertext CT connected with the attribute set.
- KeyGen (*A*, MK) → SK: The key generation algorithm takes two inputs, the access structure *A* and the master secret key MK to output a private key SK connected with the attributes.
- Decrypt (CT, SK) → *M*: The decryption algorithm also takes two inputs, ciphertext CT connected with attribute set S and a private key SK connected with access structure *A* to output a message *M*.

The strict access control policies are executed at the diagnostic centre based on the user's attributes, i.e., doctors can gain access to the entire personal medical information, the nurses/technicians can gain access only to a few medical data of the patient, and the patients and family members have very limited access to data. The medical data are available only to the authorised users and the unauthorised users cannot access the data.

## 5 Experimental results

SHS is implemented using the Net Beans IDE over the dataset referred in [14]. The performance comparisons are carried out on the basis of encryption time, decryption time and total computation time for the algorithms in [5, 6, 10].

Table 4 shows the encryption time for combinations of different algorithms. Among all the four methods, Paillier cryptosystem and digital signature consume more encryption time [6] as they split the data and do encryption three times at all the three distributed servers and again they have to perform decryption. So, the decryption time and eventually total computation time are higher compared with the other algorithms.

**Table 4:** Encryption time

	51 kB	204 kB	407 kB	1531 kB	3061 kB	6122 kB	9193 kB
Paillier cryptosystem and digital signature, s	25	55	156	235	355	456	560
AES and MD5, s	1	2.5	3.7	9	12	19	24
CP-ABE and AES, s	3.5	4.5	6.5	13	15	24	30
blowfish and CP-ABE, s – SHS	1	1	2.5	8	9	15	20

Table 5 shows the decryption time of all the four combinations of algorithms. CP-ABE and AES have large encryption and decryption cost compared with AES and MD5 algorithms because the session key is encrypted and decrypted before encryption and decryption of actual messages. This consumes high storage and computation cost compared with AES and MD5 algorithms.

**Table 5:** Decryption time

	51 kB	204 kB	407 kB	1531 kB	3061 kB	6122 kB	9193 kB
Paillier cryptosystem and digital signature, s	100	145	255	455	655	890	1000
AES and MD5, s	4.2	6	8.8	9	11	15	17.6
CP-ABE and AES, s	7.6	8.9	10.9	13.5	15	18	23
blowfish and CP-ABE, s – SHS	2.5	3.7	4.5	5.6	6.6	7.5	8.6

Table 6 shows the total computation time of the algorithms. Blowfish and CP-ABE algorithms used in SHS have very less computation time compared with the other algorithms. The medical data need to reach quickly to the destination side, i.e. to the doctors and medical personnel. As the blowfish algorithm has very less computation cost, it is better than any other combination of algorithms. At the same time, the access control is also provided to the users by executing CP-ABE algorithm. The data privacy and data access control are provided to medical data in SHS with the combination of blowfish and CP-ABE algorithms.

**Table 6:** Total computation time

	51 kB	204 kB	407 kB	1531 kB	3061 kB	6122 kB	9193 kB
Paillier cryptosystem and digital signature, s	200	250	456	750	1100	1435	1600
AES and MD5, s	7	10	15	19	25	35	42
CP-ABE and AES, s	12	14	18	30	33	42	56
blowfish and CP-ABE, s – SHS	5	5.5	7.6	13	15.2	24	30

## 6 Conclusion

The medical sensor senses the patient's physiological data and transmits them over the wireless channels which are more susceptible than wired networks. The public key algorithms are more computationally intensive than symmetric key algorithms. Moreover, they are not suitable for sending short messages. So, the symmetric key algorithms like AES, DES, blowfish, RC6 and IDEA are compared of their performance. The performance of blowfish algorithm is good, so it is used for encrypting medical data. The data must be accessed only by the authorised users with specific control and so the CP-ABE is executed. The combination of these two algorithms is suitable for healthcare applications as it provides security and fast transmission of medical data than the other existing systems.

## 7 Funding and declaration of interests

None declared.
